# Comparing machine learning approaches to incorporate time-varying covariates in predicting cancer survival time

**DOI:** 10.1038/s41598-023-28393-7

**Published:** 2023-01-25

**Authors:** Steve Cygu, Hsien Seow, Jonathan Dushoff, Benjamin M. Bolker

**Affiliations:** 1 School of Computational Science and Engineering, Hamilton, McMaster University, 1280 Main St W, Hamilton, ON L8S 4L8 Canada; 2 Department of Oncology, Hamilton, McMaster University, 1280 Main St W, Hamilton, ON L8S 4L8 Canada; 3 Department of Biology, Hamilton, McMaster University, 1280 Main St W, Hamilton, ON L8S 4L8 Canada

**Keywords:** Computational science, Breast cancer, Cancer models, Gastrointestinal cancer, Lung cancer

## Abstract

The Cox proportional hazards model is commonly used in evaluating risk factors in cancer survival data. The model assumes an additive, linear relationship between the risk factors and the log hazard. However, this assumption may be too simplistic. Further, failure to take time-varying covariates into account, if present, may lower prediction accuracy. In this retrospective, population-based, prognostic study of data from patients diagnosed with cancer from 2008 to 2015 in Ontario, Canada, we applied machine learning-based time-to-event prediction methods and compared their predictive performance in two sets of analyses: (1) yearly-cohort-based time-invariant and (2) fully time-varying covariates analysis. Machine learning-based methods—gradient boosting model (gbm), random survival forest (rsf), elastic net (enet), lasso and ridge—were compared to the traditional Cox proportional hazards (coxph) model and the prior study which used the yearly-cohort-based time-invariant analysis. Using Harrell’s C index as our primary measure, we found that using both machine learning techniques and incorporating time-dependent covariates can improve predictive performance. Gradient boosting machine showed the best performance on test data in both time-invariant and time-varying covariates analysis.

## Introduction

Early diagnosis and accurate prognosis can improve the clinical management of cancer patients. Good prognostic tools can help in treatment planning^1^, aid communication with patients and patients’ decision-making about surgery and treatments; and also help in timely and effective symptom management^2^. Measuring cancer patients’ well-being is significant in assessing response to treatment and capabilities for various types of care^3^. For instance, integrating palliative care interventions with oncological care for advanced cancer patients can lead to improved quality of life, reduced symptom burden, fewer hospital visits, and reduced health costs^1,4,5^. Predictive computational methods that predict symptoms, patients’ well-being, and survival time can help clinicians customize treatment regimes and give timely interventions.

Traditional statistical methods such as Kaplan–Meier and Cox proportional hazards (coxph) models have been used to model survival data^5–7^. Both techniques estimate the probabilities of survival past a given time. As suggested by their name, coxph models make a proportional hazards assumption—i.e. they assume an additive, linear relationship between the predictors and the log hazard^8^. In clinical survival data, especially when applying machine learning (ML) techniques, a number of challenges have been identified^9–11^: first, difficulty in dealing with censored data (i.e., time-to-event is imperfectly observed); second, although ML techniques have often proven to be effective when the number of predictors is large, this is not always the case in survival analysis^12^; and third, studies on the accuracy of clinical prediction of survival time have found poor agreement with the actual survival times, with practitioners’ predictions tending to be longer than actual survival times^5,13,14^.

Additional challenges in accurate prediction of survival of cancer patients emerge from the growing complexity of cancer, various treatment options, heterogeneous patient populations and failure to account for measurements which change over time (time-varying covariates)^5^. In survival data, time-varying covariates are common. For example, cancer patients’ chemo-therapy treatment plan or healthcare access may change over the course of the study. The standard coxph model assumes that the covariates are time-invariant and have a constant linear effect over the entire follow-up period^15,16^. Time-invariant coxph models have been extended to handle time-varying covariates^17^.

The use of ML methods in predicting the risk of death of cancer patients from clinical data is not new^11,18–20^. Depending on how these methods are applied, they can be considered standard ML methods (directly applied to predict the outcome of interest such as survival status) or ML methods for survival analysis (modified to handle time-to-event data). Standard ML methods use binary classification to predict the survival status of subjects within a particular time window. Since binary classifiers consider only whether or not the event occurred in the last observation window, they lack the interpretability and flexibility of models that consider hazards as a function of time^15^. Most ML methods for survival analysis, such as artificial neural networks (ANN)^21^, survival trees and random forest^12,22^, predict events of interest using covariates measured at the time of diagnosis, not accounting for the time-varying covariates. Furthermore, only a few of these models have incorporated patient-reported cancer diagnosis related outcomes such as level of pain as potential covariates to build predictive models^5^.

In this paper, we build, validate and compare traditional coxph models and ML models for survival analysis, using both time-invariant and time-varying covariates (including both clinical and patient reported variables). We compare the performance of our models to the prior yearly-cohort-based time-invariant traditional coxph model with backward variable selection implemented by Seow et al.^5^.

## Results

### Performance of the machine learning models

**Figure 1:**
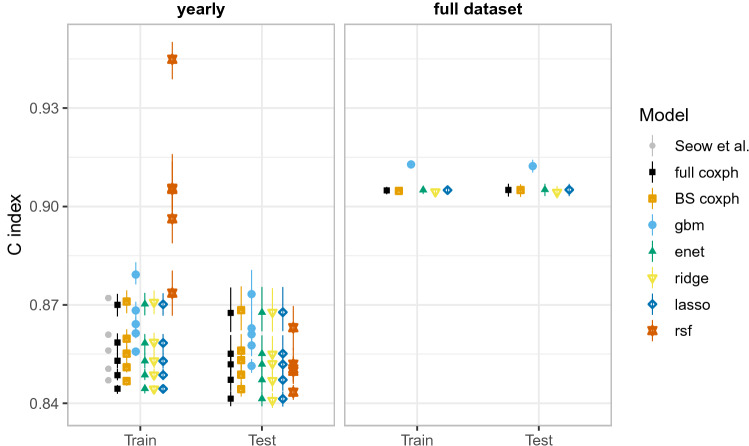
A comparison of Harrell’s concordance scores (*C* index) for the yearly-cohort-based time-invariant (yearly) and fully time-varying (full) covariates analysis. Higher values are better. The yearly-cohort-based estimates obtained in Seow et al.^5^ were available for training data only. For comparison, we provide both training and test *C* index scores. Generally, gradient boosting machine slightly performs better than all the other models.

The results of our comparisons on training and testing data sets are shown in Fig. 1, summarized by the 2.5%, 50% and 97.5% quantiles of the estimated Harrell’s *C* index on 200 bootstrap resamples of the respective data sets. The details of these comparisons are given in the Model evaluation and comparison section. The ML algorithms we used fall into 3 groups: penalized Cox model, i.e., elastic net (enet), lasso and ridge; gradient boosting machine (gbm); and random survival forest (rsf). We also compare with the traditional (non-ML) coxph model used by Seow et al.^5^, i.e., traditional coxph model with backward variable selection (BS coxph) as well as full traditional coxph (full coxph). Due to computational and implementation constraints, rsf was not implemented on the fully time-varying covariates analysis.

On the yearly-cohort-based time-invariant analysis, gradient boosting machine (gbm) had the highest score in the test data across all the cohorts. For the training data, the BS coxph method matches the earlier results almost exactly (as expected). Random survival forest (rsf), although trained on a subset of the data (5000 cases), had the highest score on the training data but performed comparably to the other models (except gbm) on the testing data.

On the fully time-varying covariates analysis, gbm’s predictive performance was again higher than all other models, on both training and testing data. The performance of the other models (including the traditional models) was similar. The models were generally able to achieve better prediction when using the fully time-varying covariates than when using yearly-cohort-based time-invariant covariates. Separate cohort comparisons are provided in Supplementary Fig. S1.

### Temporal performance of the models

To evaluate the performance of the models at different survival marks, we use the time-dependent AUC. We compare the year 0 cohort analysis (which combines all the cohorts, using their covariates at time 0) to the full dataset (using time-varying covariates). The difference between panels thus directly quantifies the effect of incorporating time-varying covariates.

**Figure 2:**
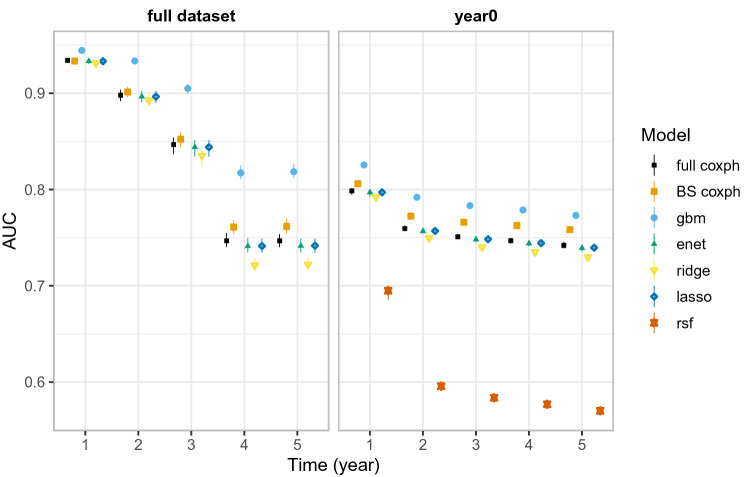
Time-dependent AUC scores evaluated at different time points. The scores are based on 200 bootstrapped samples (50 for rsf due to computational limitations) of the test data. Models with higher scores and narrower confidence intervals are better performers. As with the concordance index, models do better with the fully time-varying covariates (full), and gbm does better than other models.

Figure 2 shows the distributional summary of the time-dependent AUC achieved in 200 replicates of bootstrapped samples of the test data. The central point represents the median (50%) quantile, while the lower and upper ends of the lines represent lower (2.5%) and upper (97.5%) quantiles of the estimates. Higher values indicate better performance, narrower ranges indicate more stable algorithms.

In both fully time-varying and yearly-cohort-based time-invariant covariates, gbm model has slightly better estimates with comparatively narrower confidence intervals. Estimates based on the fully time-varying covariates are generally better than estimates based on the yearly-cohort-based time-invariant covariates.

### Most prognostic predictors

**Figure 3:**
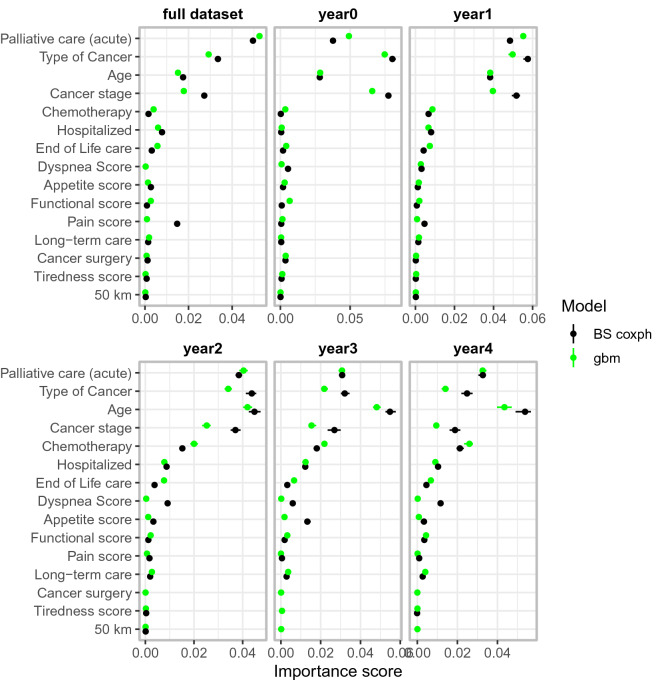
Variable importance scores together with the corresponding 2.5%, 50% and 97.5% quantiles, based on the best (gbm) and benchmark models (BS coxph). Notably, palliative care, age of the patient, cancer type and cancer stage stood out, across the cohorts, as some of the most important prognostic factors on survival of cancer patients.

Figure 3 shows permutation-based importance scores (see “Methods”) for the top 15 features in each data set for both gbm (the top performing model), and for BS coxph (for the benchmark model). Palliative care, cancer type, age and cancer stage were identified as important in all cases.

**Figure 4:**
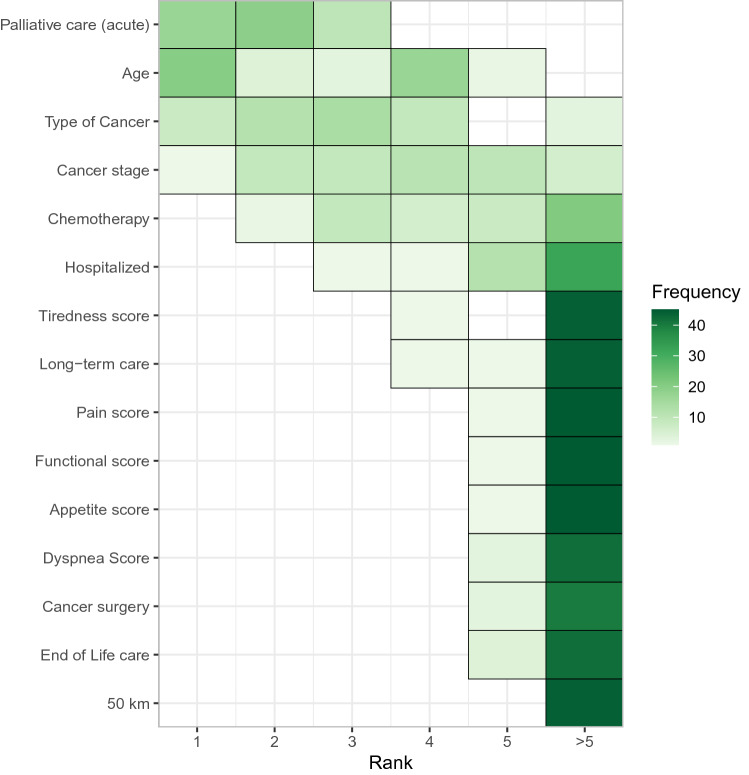
The number of times, frequency, a given feature is ranked, on top 5, by a particular model in a given cohort as one of the most important feature. Low rank means a particular feature is predictive and hence important.

To compare the features across the cohorts, we ranked all the features and counted the number of times each feature was among the top 5 across all the models and cohorts (Fig. 4).

## Discussion

Compared to most machine learning algorithms, traditional coxph models are less suited for prediction, although the full (unselected) coxph may be better for inference about the impact of a specific predictor. In our analyses the rsf was unusual among ML models in performing badly, although it is particularly well suited to measuring the predictive importance of input variables. If our interest is to predict time-to-event of cancer patients based on a number of clinical, medical and self-reported predictors, machine learning-based models may be preferable over traditional coxph models in analyses that consider more data at once.

Fitting models which incorporate fully time-varying covariates require special attention; only a subset of the models fitted in the yearly-cohort-based time-invariant models could support this kind of analysis. Thus, in the full data set incorporating time-varying covariates, in addition to traditional coxph, only gradient boosting and penalized models were implemented.

Harrell’s *C* index was measured on mutually exclusive training and testing data sets. For overfitted models, we would expect the model to perform well on the training set but poorly on the testing set. Except for rsf, none of the models appear overfitted. Penalized models did not show major improvement in predictive performance over the traditional coxph model with backward variable selection model which was slightly better than the full traditional coxph model.

Our results show that ML-based methods can provide more accurate alternatives to traditional hazard-based methods in both yearly-cohort-based time-invariant and full time-varying covariates. However, coxph model with backward variable selection performed comparably to the ML-based methods, as has also been seen elsewhere^5,23^. Cox model with gradient boosting machine had the highest predictive performance score in all the comparisons done. This model has additional advantages of computational efficiency and fewer hyperparameters when compared to methods like random survival forests models. In particular, tuning hyperparameters for random forest, even using a subset of the training data, was not straightforward and took a considerable amount of time. We also tried training neural network models but were not successful due to difficulty in tuning of hyperparameters and computational limitations.

In summary, time-varying covariates greatly improve model prediction, and not only in the ML context. We also find that gradient-boosting machine (gbm) improves performance across both the cohort and time-varying approaches, suggesting that it may be a good choice in general for problems of this nature.

## Methods

### Study participants

Subjects were adults diagnosed with cancer from a population-based, retrospective prognostic study, as confirmed by the provincial cancer registry in Ontario, Canada, from January 1, 2008, to December 31, 2015.

The study was reviewed by Hamilton Integrated Research Ethics Board and deemed exempt because it used de-identified secondary data.

Patients and the public were not involved in this research. It used de-identified, secondary administrative data analysis, (which is allowed to be used for research purposes), and thus patient consent was not obtained. Seow et al.^5^ provide a detailed description of the data and study setting.

### Data pre-processing

A number of pre-processing steps were undertaken to prepare the data set for modelling. To avoid excluding cases or variables from the data set, a “missing” category was created for the patient-reported categorical variables. Numerical variables, such as age, were mean-centered.

### Analysis plan

**Figure 5:**
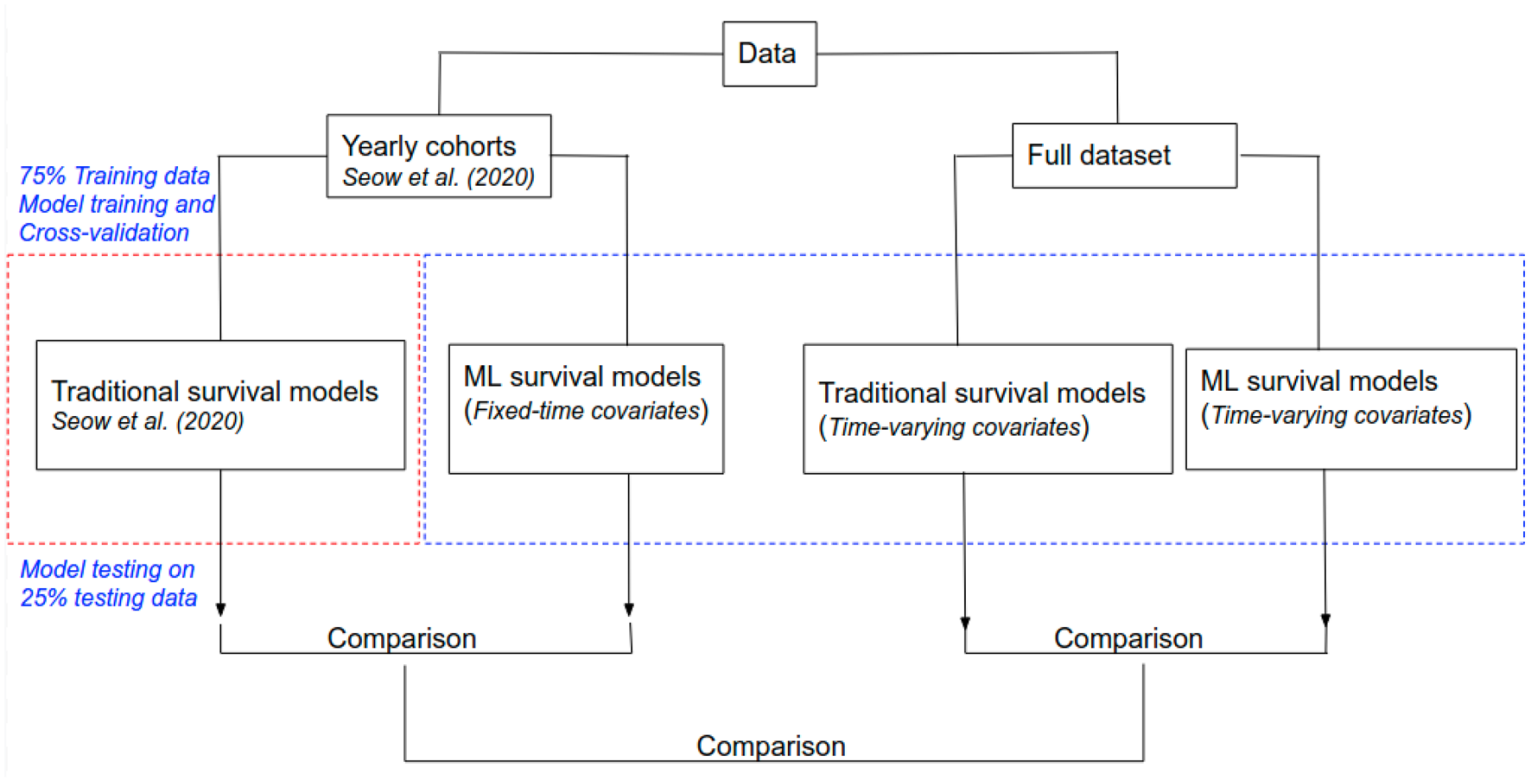
The blue dotted rectangle indicates the major analytical contribution of this paper. We also replicated analyses by Seow et al.^5^, indicated by red rectangle. We performed two classes of analysis depending on the nature of the data set. The first set of analyses closely followed modelling procedure in Seow et al.^5^ which is based on yearly cohorts (we refer to these as yearly cohort models) and, by construction, takes care of changing covariates over the observation period. We then used ML models for survival analysis and compared predictive performance to prior results in Seow et al.^5^, which used traditional coxph model with backward variable selection. The second set of analyses used both traditional coxph and ML models for survival analysis which directly incorporate time-varying covariates on the full data set. We refer to these as full data set models. We also compared predictive performance of the full data set models to those of yearly cohort models.

We performed two classes of analysis which were based on the structure of the data, *yearly cohort* (yearly-cohort-based covariates) and *full data set* (fully time-varying covariates), as summarized in Fig. 5.

#### Yearly cohort models

**Figure 6:**
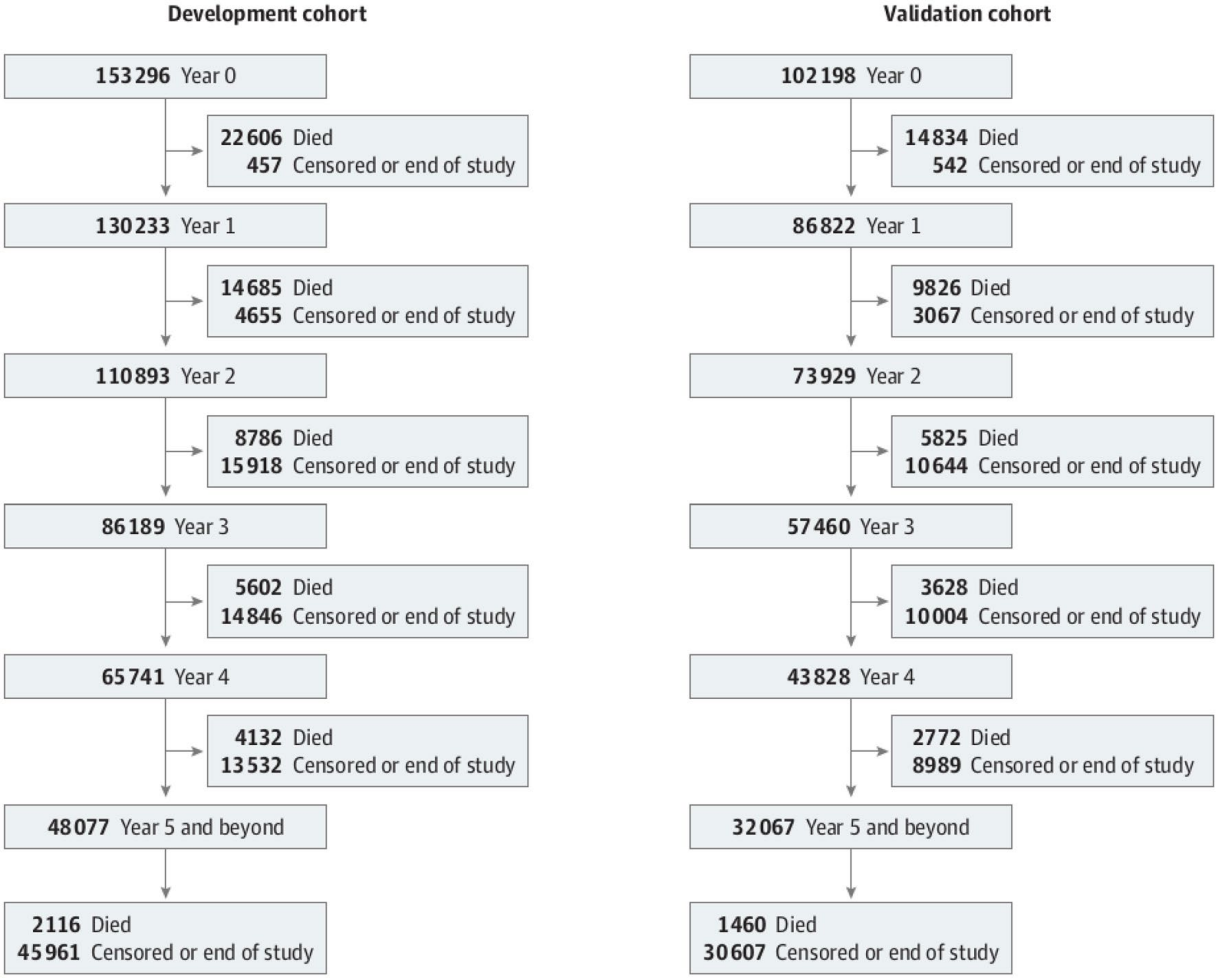
The yearly cohorts defined by Seow et al.^5^.

To account for changing covariates in a traditional coxph model, Seow et al.^5^ created yearly cohort data sets. Each year that a patient survived post-diagnosis, they were entered into a separate cohort model; thus, only patients who survived to a certain point (survival mark) contributed to the corresponding conditional analysis, and their survival times were adjusted to reflect this conditioning. For example, for an individual who survived for 2.5 years: the Year 0 cohort contains baseline covariate information and a survival time of 2.5 years; the Year 1 cohort contains the current covariate information and a survival time of 1.5 years; and Year 2 cohort contains updated covariate information and a survival time of 0.5 years. Cohort definitions are summarized in the Fig. 6. Our first set of ML models used these yearly cohort data sets (rsf used a random sub-sample of 5000 cases of each yearly cohort data set), and compared the predictive performance with those obtained from traditional coxph models fitted in the prior analyses by Seow et al.^5^. In addition to our ML fits, we also replicated the traditional coxph model together with the backward variable selection procedure in Seow et al.^5^ with slight modifications. For instance, our models used $75 - 25\%$ as opposed to $60 - 40\%$ *train–test* partition.

#### Full data set models

Both traditional coxph and ML models for survival analysis which incorporate time-varying covariates require the data set to be in a specific format—that is, counting process format^24–26^. The data are expanded from one record-per-subject to one record-per-interval between each event time, per subject, such that each record corresponds to the interval (365 days in this case) of time during which the entries of time-varying covariates are treated as constant. Once this special data set, which combines all the yearly cohorts, has been constructed, the event time is now defined by $(\textrm{start}, \, \textrm{stop}]$ interval during which the subject was continuously at risk of the event. For example, for an individual who survived for 3 years, the “at-risk” interval is defined as (0, 365], (365, 730] and (730, 1095], representing the segments in which they are event free and uncensored.

The difference between full data set and Year 0 cohort is that, in the former, the covariate information for every surviving patients is updated at each “at-risk” interval, while the latter contains the baseline covariate information about the patients. The updated, baseline-like (year1/2/3/4) cohorts from the original work are in fact useful: they show what predictions might have been made from updated covariate information at a given time. Analyzing how such predictions could be improved is therefore also of interest to us.

Currently, only penalized, gradient boosting machine and random forest implementations support time-varying covariates. Due to computational challenges, we only fitted and compared penalized, i.e., lasso, ridge and elastic net and gradient boosting machine models, in addition to the traditional coxph model. All computations were carried out on a server with 4 clusters, each with 8 Intel Xeon 3.40 GHz CPUs and a 128 GB RAM.

### Prediction methods

In addition to traditional Cox proportional hazard modeling, three machine learning algorithms capable of handling censored data were used in this analysis. The outcome of interest was time to death (days) as recorded in the Vital Statistics database^5^. The following classes of models were trained and evaluated:

1. Traditional coxph model with backward variable selection (BS coxph) and full traditional coxph (full coxph): Implemented for both time-invariant and time-varying covariates.
2. Cox-based gradient boosting machine (gbm): Implemented for both time-invariant and time-varying covariates.
3. Penalized Cox model: elastic net (enet), lasso and ridge. Implemented for both time-invariant and time-varying covariates.
4. Random survival forests (rsf): Capable of handling both time-invariant and time-varying covariates but requires large amount of computer memory for large data sets due to large forests constructed during model training. Due to this challenge, we trained random forest on only a subset of data (5000 cases) for each cohort in the time-invariant covariates analysis.

Table 1 provides a summary of models which were trained on yearly cohorts or full (time-varying) datasets. A brief description of these algorithms can be found in the Supplementary Methods S1.

**Table 1:** A summary of trained models.

Model	Sub-models	Yearly cohorts	Full dataset (time-varying)
Cox proportional hazards (coxph)	Full	Yes	Yes
	Backward selection	Yes	Yes
Gradient boosting machine (gbm)		Yes	Yes
Penalized Cox regression	Lasso	Yes	Yes
	Ridge	Yes	Yes
	Elastic net (enet)	Yes	Yes
Random survival forests (rsf)		Yes	No

Each of the ML algorithms outlined above has at least one hyper-parameter and, as result, requires parameter tuning. For this, we perform 10-fold cross validation. For penalized approaches (lasso, ridge and elastic net), the hyper-parameters are tuned using cross-validated partial log-likelihood; for random survival forest and gradient boosting machine, Harrell’s concordance index *C* is used. For the Cox proportional hazard model, we apply stepwise variable elimination on the multivariate model which fits all the covariates and identifies a subset of important variables according to Akaike’s information criterion. The final coxph model is then fitted using only those variables selected in the stepwise procedure. A list of hyper-parameters that were tuned can be found in Supplementary Table S1, together with the R packages used to implement each of the models.

### Model evaluation and comparison

The models implemented in this work have different strengths and limitations in terms of assumptions, interpretability, computational efficiency, etc. In this work, we focus on comparing the predictive accuracies of these models. We implemented the following metrics to evaluate and compare the performance of our models on the test data:

- *Harrell’s concordance index (C* *index)*. In survival analysis, a pair of patients is called concordant if the risk of the event predicted by a model is lower for the patient who experiences the event at a later time-point. The concordance index is the frequency of concordant pairs among all comparable pairs of subjects. Pairs are incomparable if their event times are equal, or if either subject is censored before the other subject experiences an event^27^. Harrell’s *C* index can be used to measure and compare the discriminative power of a risk prediction models^28^. It provides a holistic measure of the model performance over the entire time period, while allowing for censoring.
- *Time-dependent AUC*. The Receiver Operating Characteristic (ROC) curve and the associated area under curve (AUC) are widely used in medical research to quantify the discriminating power of machine learning models. The ROC curve plots the probability of both true positive (proportion of positive class correctly classified by the model) and the false positives (proportion of the negative class incorrectly classified by the model) at various cut off values of the risk score. The AUC summarizes the probabilities of true and false positives over all possible cut off values into a value ranging between 0 and 1; and gives an overall measure of predictive accuracy of a predictive model. The standard ROC considers the event status and the risk scores as fixed over time; however, in many medical applications, these quantities may change over the follow-up time; in such situations, binary classification of cases (as true positive and true negative) without taking into account the time-to-event may be inappropriate. Heagerty et al.^29^ proposed a time-dependent ROC which extends the standard ROC curve analysis for binary outcome data to time-to-event data (see Supplementary Methods S2).

To evaluate the sensitivity and uncertainty of the predictive performance measures, we applied bootstrap resampling to estimate the 2.5%, 50% and 97.5% quantiles of the distribution of the scores. We used 200 bootstrap resamples of both training and test data sets. The training estimates were included for comparison with those reported in Seow et al.^5^.

### Model validation and prediction

For the yearly-cohort-based time-invariant analysis, the model validation is similar to that by Seow et al.^5^. In particular, the models are derived using the corresponding yearly cohort training data, and then validated on the test data. However, as opposed to Seow et al.^5^ analysis which predicts the 1-year probability of death on the test data using an alternative estimator of concordance index for right-censored data based on inverse probability of censoring weights (inverse probability of censoring weighted [IPCW] *C* index)^30^, this study uses Harrell’s *C* index^27^ which evaluates the model’s performance over the entire follow-up period using the current covariates for individuals in the risk set when the first event occurs; and is as well used for the time-varying covariates models, i.e., full analysis. The existing implementations of the IPCW *C* index do not extend to analysis of time-varying covariates. Hence, for comparison purposes, we report Harrell’s *C* index for the time-invariant and time-varying covariates analysis as shown in Fig. 1. We also include comparisons based on the IPCW *C* index for the time-invariant covariate models in Supplementary Fig. S2. For the yearly cohort models, all the time-varying covariates are adjusted at each new survival mark to avoid incorporating time-varying covariates into the models. Similarly, for the full dataset models, the information about the covariates is updated until the current follow-up time, as previously explained. This allows predictions to be based on current information about the covariates, and not beyond what is observed.

Table 2 provides a summary of model evaluation metrics for yearly-cohort-based time-invariant and full (time-varying) analysis, together with the corresponding figures.

**Table 2:** Evaluation metrics.

Metric	Model	Yearly cohorts	Full (time-varying) dataset	Figures
Harell’s C index	All models	Yes	Yes	Figs. 1 & S2
Time-dependent AUC	All models	Yes	Yes	Fig. 2
IPCW C index	All models	Yes	No	Fig. S2

### Identifying prognostic features

A permutation-based variable importance score was used to identify the most important prognostic features. For each replicate, we randomly resample the values of a focal predictor and record how our metric changes due to this perturbation. The key idea is that if a particular predictor has high power to predict the response, then randomly permuting its observed values will lead to a considerable change in the predictive accuracy of the model. In this case, we conclude that this predictor is important. In our implementation, Harrell’s *C* index is used as a measure of predictive power.

### Limitations

Some limitations of this study are related to the shortcomings in the available data, as discussed in Seow et al.^5^. In particular, information about performance status and symptoms at various follow-up times were missing. Biomarker and targeted therapies data were not available; this auxiliary information could improve the predictive accuracy of the models. As previously mentioned, due to memory limitations, the random survival forest model was trained on a subset of the data. Moreover, even on this small subset of data (5000 cases), we could not properly tune hyperparameters for the neural network models (not presented here) using the computational resources available within our computational platform, which was specialized for hosting sensitive data.

## Supplementary Information

Supplementary Information.

## Data Availability

The de-identified administrative data are not publicly available and may be obtained from a third party, ICES (formerly the Institute for Clinical Evaluative Sciences) for researchers who meet the criteria for permissible access. These data represent secondary data analysis and are not owned or collected by the study authors. A data request can be sent here: https://www.ices.on.ca/About-ICES/ICES-Contacts-and-Locations/contact-form. We provide all the R codes used for the analysis in the form of a workflow R package for the analysis of similar data sets, which can be accessed on GitHub through https://github.com/CYGUBICKO/satpred.
