# Supplementary material for: Comparing machine learning approaches to incorporate time-varying covariates in predicting cancer survival time
Source: Sci Rep. 2023 Jan 25;13:1370. doi: 10.1038/s41598-023-28393-7 (PMC9877029; doi:10.1038/s41598-023-28393-7)
Supplement: Supplementary file 1 — Supplementary Information. [file 41598_2023_28393_MOESM1_ESM.pdf]

Comparing machine learning approaches to incorporate time-varying covariates in predicting cancer survival time

Steve Cygu[1,*], **Hsien Seow**[2], **Jonathan Dushoff**[1,3], **and Benjamin M. Bolker**[1,3]

[1]McMaster University, School of Computational Science and Engineering, Hamilton, 1280 Main St W, Hamilton, ON L8S 4L8, Canada
[2]McMaster University, Department of Oncology, Hamilton, 1280 Main St W, Hamilton, ON L8S 4L8, Canada
[3]McMaster University, Department of Biology, Hamilton, 1280 Main St W, Hamilton, ON L8S 4L8, Canada
[*]cygubicko@gmail.com

Supplementary Methods S1: Machine learning algorithms

Standard machine learning methods use binary classification to predict whether an event occurs within a specified time window. However, such binary classifiers fail to account for the censored observations. Several ML algorithms have been extended to estimate hazards and use a survival-model paradigm to consider event times (and handle observation censoring). Below, we summarize the traditional Cox proportional hazards model approach and give a short description of some ML models for survival analysis.

Cox proportional hazards model (coxph)

Time-invariant covariate survival data is often presented in the form $\{t_i, \delta_i, x_i\}_{i=1}^n$, where t_i is the observed event time for individual i, δ_i is an indicator variable for censored or observed event of interest, and x_i is a vector of covariates. Traditional hazard-based methods such as Cox proportional hazards (coxph) model[1] is commonly used in survival data. The coxph model defines the hazard function at time t as

$$h_i(t) = h_0(t)\exp(x_i^\top \beta), \tag{1}$$

where $h_0(t)$ is the non-parametric baseline hazard function and $\exp(x_i^T \beta)$ is the relative hazard, which summarizes the effects of the covariates[2]. Under the proportional hazards assumption, the model parameters can be estimated by minimizing a partial log-likelihood which does not involve the non-parametric baseline hazard.

When covariates change over time during the follow-up period, the observed survival data is of the form $\{t_i^{\text{start}}, t_i^{\text{stop}}, \delta_i, x_i(t)\}_{i=1}^n$. The only difference is that x_i is now a (piecewise constant) function of time, and Equation 1 adjusted appropriately for downstream implementation[3,4].

Although coxph models are commonly used, especially when the main goal is to make inference on how the covariates impact on the survival probabilities. However, in prediction context, ML models for survival analysis may be desirable.

Penalized cox proportional hazards models

Traditional coxph models may overfit, particularly in the case of high-dimensional data[2]. Penalized methods such as **lasso**, **ridge** and **elastic net** offer a convenient way of addressing overfitting. Lasso and elastic net can also be used to select a subset of useful predictive feature while eliminating others.

Penalized methods add a penalty to the log-likelihood function, which has the effect of shrinking the coefficient values towards zero, reducing sampling variance and reducing the impact of less important features on the model. The ℓ_1 (lasso) penalty is based on the absolute value of the coefficients. It typically

reduces the number of predictors used (by assigning zero coefficients to a subset of predictors). The number of features selected by lasso is bounded by number of observations[5]. On the other hand, ℓ_2 (ridge) penalty is based on the square of the coefficients. The ridge penalty shrinks the coefficients towards (but never all the way to) zero. The elastic net (a combination of ℓ_1 and ℓ_2) penalties combines the strength of lasso and ridge for improved predictive performance[2].

Lasso, ridge and elastic net regression have all been extended to handle time-varying covariates survival data and are evaluated here. See Cygu et al.[3] for a detailed discussion on penalized Cox proportional hazards model for time-dependent covariates.

Random survival forest (rsf)

Random forests are ensembles of decision trees that are grown on bootstrapped training samples of the original data by choosing *m* random samples of the original set of *p* predictors at each split (node). Random survival forests (RSF) are random forests adapted for survival analysis of censored data. In a random survival forest, the feature and split point chosen is the one that maximizes the survival difference between daughter nodes, i.e., that maximizes the log rank statistic over all available split points and features[6]. As opposed to coxph-based approaches, which assume linear combination of the covariates, RSF are capable of automatically handling and identifying non-linear and complex interactions. A RSF is free of assumptions; and due to the randomization during splitting, it can perform feature selection through measures of variable importance. A possible drawback of RSF is the bias in splitting in the presence of predictors with multiple possible split points, e.g., categorical predictors with many levels[7]. In our implementation, we ran into computational memory issues due to large forests constructed in model training. As a result, we trained RSF on only a subset of the time-invariant cohorts data set. A detailed description of RSF is outlined in Ishwaran et al.[6]. We used the following hyperparameters for our random survival forest fits:

- The number of trees to grow, **ntree**.
- The number of variables randomly selected for splitting a node, **mtry**.
- Minimum size of a node (after splitting), **nodesize**.
- Maximum depth to which a tree should be grown, **nodedepth**. A *NULL* value grows individual full trees without pruning.
- The rule for splitting nodes, **splitrule** (log rank or log rank score).

Yao et al.[8] generalized the conditional inference and relative risk survival forests to incorporate time-varying covariates and proposed a more general framework for estimating survival function in the presence of time-varying covariates. However, due to computational limitations, we did not implement this method.

Generalized boosted regression models (gbm)

Boosting is an iterative method which uses an ensemble technique to train weak learners sequentially, where each new model that is added to the ensemble learns from the "mistakes" of the previous models.

There are two main approaches to boosting in survival analysis: likelihood-based and gradient boosting. Likelihood-based boosting uses base learners that maximize the overall likelihood in each boosting step, selecting only the base-learner which leads to largest increase in the likelihood. On the other hand, gradient boosting is equivalent to iteratively re-fitting the residuals of the ensemble model at each step. With correct

choice of boosting steps, boosted models are resistant to overfitting and work well in high-dimensional data[7]. In this work, we used gradient boosting machine with the following hyperparameters:

- The number of trees, **n.trees**.
- The shrinkage parameter, **shrinkage**, which controls the rate at which boosting learns. Small values of shrinkage require using large values of n.trees.
- The interaction depth, **interaction.depth**, which controls the complexity of the boosted ensemble, i.e., the highest level of variable level interaction. A value of 1 implies an additive model, a value of 2 implies a model with up to 2-way interactions, etc.

Supplementary Methods S2: Model evaluation methods

Time-dependent AUC

Let R be the estimated or predicted risk score, T denote the time to the occurrence of the event of interest and t define some time horizon. The individual's event status at t is defined as $D(t) = 1\{T \leq t\}$, which equals 1 if the event has occurred and 0 otherwise. Assuming that a higher value of R is associated with higher risk of event occurrence and that individual is predicted to have experienced event in the interval $(0,t]$ if $R > c$, where c is some cut off value, otherwise the individual is predicted to be event free in the interval $(0,t]$. Heagerty et al.[9] defined the sensitivity and specificity at the time horizon t as

$$\begin{aligned} \text{Sensitivity}(c,t) &= P(R > c|T \leq t) \\ \text{Specificity}(c,t) &= P(R \leq c|T > t). \end{aligned} \tag{2}$$

The major difference between the definition of sensitivity and specificity in standard binary case and the definition in Equation 2 is that the latter is defined with respect to the time horizon t. Sensitivity(c,t) and Specificity(c,t) are referred to as time-dependent sensitivity and specificity, respectively, and the resulting ROC curve (plot of Sensitivity(c,t) against $1-$Specificity(c,t)) is the time-dependent ROC curve at time horizon t. For our analysis, we used **R** software package **risksetROC** which implements this extension[10, 11].

Supplementary Figure S1: Harrell's C-index for each cohort

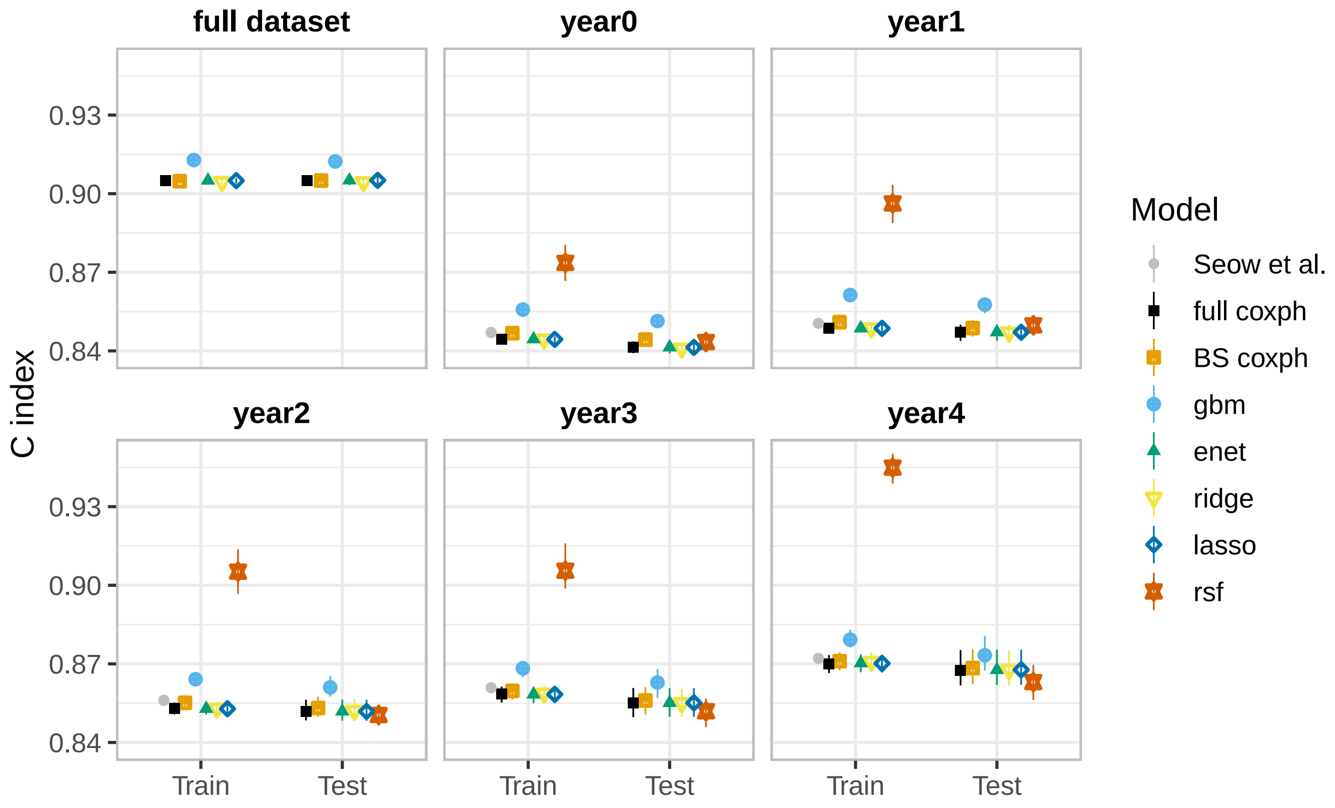

Figure 1. A comparison of Harrell's concordance scores (*C* index) for the full data set and the yearly cohorts. Higher values are better. For comparison with Seow et al.[12], we provide both training and test *C* index scores. Generally, gradient boosting machine slightly performs better than all the other models. This a different way to present Figure 1 shown in the Results section, i.e., it compares the scores within the full data set and yearly cohorts.

Supplementary Figure S2: IPCW C index

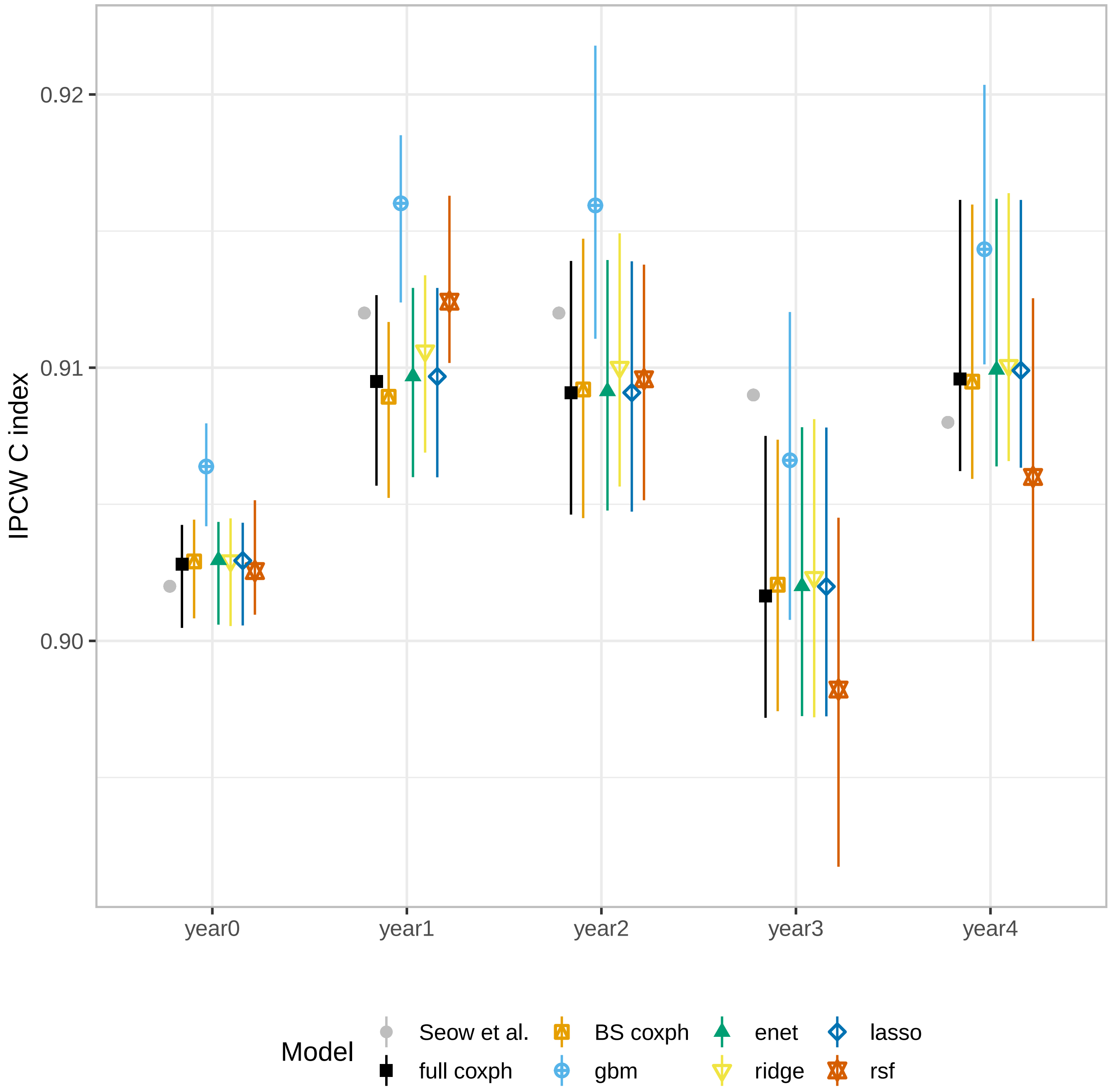

Figure 2. A comparison of IPCW C indices for the yearly cohorts. Higher values are better. For comparison with Seow et al.[12], we provide scores for accuracy for the 1-year survival probability. In general, the gradient boosting machine performs slightly better than all the other models. The current implementations of the IPCW C index do not support time-varying covariate analysis. There are no major variations in Seow et al.'s[12] and our implementation of yearly cohorts; the patterns of model performance quantified by the IPCW C index are similar to the ones generated using Harrell's C index.

Supplementary Table S1: Tuning parameters

Models	R package	Data	Tuned hyper-parameters
Cox PH model; backward selection	survival; rms	All	
Ridge	glmnet	Year 0	alpha=0, lambda=0.0250
		Year 1	alpha=0, lambda=0.0238
		Year 2	alpha=0, lambda=0.0184
		Year 3	alpha=0, lambda=0.0162
		Year 4	alpha=0, lambda=0.0147
		Full	alpha=0, lambda=0.0203
Elastic Net	glmnet	Year 0	alpha=0.4, lambda=0.0024
		Year 1	alpha=0.8, lambda=0.0011
		Year 2	alpha=0.6, lambda=0.0011
		Year 3	alpha=0.8, lambda=0.0006
		Year 4	alpha=0.2, lambda=0.0014
		Full	Alpha=0.5, lambda=0.0007
LASSO	glmnet	Year 0	alpha=1, lambda=0.0012
		Year 1	alpha=1, lambda=0.0010
		Year 2	alpha=1, lambda=0.0007
		Year 3	alpha=1, lambda=0.0005
		Year 4	alpha=1, lambda=0.0004
		Full	alpha=1, lambda=0.0004
Random survival forest	randomForestSRC	Year 0	ntree=500, mtry=15, nodesize=40, splitrule="logrank", nodedepth=NULL
		Year 1	ntree=500, mtry=10, nodesize=25, splitrule="logrank", nodedepth=NULL
		Year 2	ntree=500, mtry=10, nodesize=25, splitrule="logrank", nodedepth=NULL
		Year 3	ntree=500, mtry=10, nodesize=25, splitrule="logrank", nodedepth=NULL
		Year 4	ntree=500, mtry=10, nodesize=15, splitrule="logrank", nodedepth=NULL
Gradient boosting machine	gbm and gbm3	Year 0	shrinkage=0.1, n.trees=3548, interaction.depth=2
		Year 1	shrinkage=0.1, n.trees=3541, interaction.depth=2
		Year 2	shrinkage=0.1, n.trees=2711, interaction.depth=2
		Year 3	shrinkage=0.1, n.trees=2112, interaction.depth=2
		Year 4	shrinkage=0.1, n.trees=1443, interaction.depth=2
		Full	Shrinkage=0.1, n.trees=2982, interaction.depth=2

Table 1. Tuning parameters

References

1. Cox, D. R. Regression Models and Life-Tables. *J. Royal Stat. Soc. Ser. B (Methodological)* **34**, 187–202, DOI: 10.1111/j.2517-6161.1972.tb00899.x (1972).

2. Simon, N., Friedman, J., Hastie, T. & Tibshirani, R. Regularization Paths for Cox's Proportional Hazards Model via Coordinate Descent. *J. Stat. Softw.* **39**, DOI: 10.18637/jss.v039.i05 (2011).

3. Cygu, S., Dushoff, J. & Bolker, B. M. pcoxtime: Penalized Cox Proportional Hazard Model for Time-dependent Covariates. *arXiv:2102.02297 [stat]* (2021). ArXiv: 2102.02297.

4. Harrell, F. E. *Regression Modeling Strategies: With Applications to Linear Models, Logistic and Ordinal Regression, and Survival Analysis*. Springer Series in Statistics (Springer International Publishing, Cham, 2015).

5. Tibshirani, R. J. The lasso problem and uniqueness. *Electron. J. Stat.* **7**, DOI: 10.1214/13-EJS815 (2013).

6. Ishwaran, H., Kogalur, U. B., Blackstone, E. H. & Lauer, M. S. Random survival forests. *The Annals Appl. Stat.* **2**, DOI: 10.1214/08-AOAS169 (2008).

7. Spooner, A. *et al.* A comparison of machine learning methods for survival analysis of high-dimensional clinical data for dementia prediction. *Sci. Reports* **10**, 20410, DOI: 10.1038/s41598-020-77220-w (2020).

8. Yao, W., Frydman, H., Larocque, D. & Simonoff, J. S. Ensemble Methods for Survival Data with Time-Varying Covariates. *arXiv:2006.00567 [stat]* (2021). ArXiv: 2006.00567.

9. Heagerty, P. J., Lumley, T. & Pepe, M. S. Time-Dependent ROC Curves for Censored Survival Data and a Diagnostic Marker. *Biometrics* **56**, 337–344, DOI: 10.1111/j.0006-341X.2000.00337.x (2000).

10. Heagerty, P. J. & Zheng, Y. Survival Model Predictive Accuracy and ROC Curves. *Biometrics* **61**, 92–105, DOI: 10.1111/j.0006-341X.2005.030814.x (2005).

11. Heagerty, P. J. & packaging by Paramita Saha-Chaudhuri. *risksetROC: Riskset ROC curve estimation from censored survival data* (2012). R package version 1.0.4.

12. Seow, H. *et al.* Development and Validation of a Prognostic Survival Model With Patient-Reported Outcomes for Patients With Cancer. *JAMA Netw. Open* **3**, e201768, DOI: 10.1001/jamanetworkopen.2020.1768 (2020).
